# Solid epithelioid peritoneal mesothelioma with pulmonary metastasis in feline

**DOI:** 10.29374/2527-2179.bjvm004523

**Published:** 2024-02-28

**Authors:** Heloísa Cristina Teixeira de Carvalho, Lígia Fernandes Gundim, Felipe Martins Pastor, Gabriel Henrique Guimarães, Arlinda Flores Coleto, Matias Pablo Juan Szabó, Alessandra Aparecida Medeiros-Ronchi

**Affiliations:** 1 Veterinarian, Programa de Pós-Graduação em Ciências Veterinárias (PPGCV), Departamento de Patologia Animal (LPA), Faculdade de Medicina Veterináira (FAMEV), Universidade Federal de Uberlândia (UFU). Umuarama, MG. Brazil; 2 Veterinarian, DSc., PPGCV, FAMEV, UFU. Umuarama, MG. Brazil; 3 Veterinarian, Resident. Programa de Residência em Medicina Veterinária - Patologia Animal. FAMEV, UFU. Umuarama, MG, Brazil; 4 Veterinarian, MSc. PPGCV, LPA, FAMEV, UFU. Umuarama, MG. Brazil; 5 Veterinarian, PhD., LPA, Hospital Veterinário, UFU, Uberlândia, MG, Brazil

**Keywords:** cat, mesothelium, peritoneum, tumor, gato, mesotélio, peritônio, tumor

## Abstract

Mesothelioma is a rare malignant neoplasm that affects the mesothelial cells lining the thoracic and abdominal cavities, such as the pleura, peritoneum, and pericardium. It is most prevalent in dogs and cattle, but the causes of this disease in animals are uncertain. In felines, it mainly affects the pleura, with an unfavorable prognosis. This paper explores a rare case of metastatic peritoneal mesothelioma in a 2-year-old female mixed breed cat, emphasizing its uniqueness due to the feline's age. The patient, previously treated at a private clinic, presented moderate abdominal distension as the only clinical sign. Abdominal ultrasound and peritoneal fluid cytology led to the provisional diagnosis of mesothelioma/carcinomatosis. One day after exploratory laparotomy, the animal died and was subsequently sent for necropsy. During macroscopic analysis, nodules were observed in the peritoneum, diaphragm, omentum, stomach serosa, and large intestine, and the diagnosis of solid epithelioid peritoneal mesothelioma with lung metastasis was confirmed after microscopic analysis. The diagnosis of mesothelioma is challenging, and the importance of immunohistochemical panels with specific markers such as cytokeratin AE1/AE3 and calretinin is highlighted. Considering that mesothelioma is a pathology with a poor prognosis, it is essential to include this disease in the list of differential diagnoses within veterinary oncology.

## Resumo

O mesotelioma é uma neoplasia maligna rara que afeta as células mesoteliais que revestem as cavidades torácica e abdominal, como a pleura, o peritônio e o pericárdio. É mais prevalente em cães e bovinos, mas as causas desta doença em animais são incertas. Nos felinos acomete principalmente a pleura, com prognóstico desfavorável. Este artigo explora um caso raro de mesotelioma peritoneal metastático em uma gata sem raça definida de 2 anos de idade, enfatizando sua singularidade devido à idade do felino. O paciente, previamente atendido em clínica particular, apresentava distensão abdominal moderado como único sinal clínico. A ultrassonografia abdominal e a citologia do líquido peritoneal levaram ao diagnóstico provisório de mesotelioma/carcinomatose. Um dia após a laparotomia exploratória, o animal veio a óbito e posteriormente encaminhado para necropsia. Durante a análise macroscópica, foram observados nódulos no peritônio, diafragma, omento, serosa estomacal e intestino grosso e o diagnóstico de mesotelioma peritoneal epitelioide sólido com metástase pulmonar foi confirmado após análise microscópica. O diagnóstico do mesotelioma é desafiador, sendo destacada a importância de painéis imunohistoquímicos com marcadores específicos como citoqueratina AE1/AE3 e calretinina. Considerando que o mesotelioma é uma patologia de prognóstico ruim, é fundamental incluir esta doença na lista de diagnósticos diferenciais dentro da oncologia veterinária.

## Introduction

Mesothelioma is a malignant neoplasm of mesothelial cells that make up the serous linings of the thoracic and abdominal cavities (Uzal et al., 2016), commonly involving the pleura, peritoneum, and pericardium (Bertolini, 2017). It is rare in felines and other species, with a higher frequency in dogs and cattle (Uzal et al., 2016). The development of peritoneal mesothelioma is more common in humans and typically associated with asbestos and other particles with similar physicochemical properties (Gelberg, 2018; Munday et al., 2017). In animals, there is the hypothesis of an association between urban dogs exposed to asbestos and the incidence of mesothelioma, based on microscopic findings of ferruginous bodies in the lungs of canines (Harbison & Godleski, 1983). However, in veterinary medicine, the etiology has not been fully elucidated (Caswell & Williams, 2016; Munday et al., 2017).

The macroscopic appearance of mesothelioma varies from isolated single masses to diffuse thickening of the peritoneum. However, the most common presentation includes discrete to coalescent millimeter- to 10-cm-sized nodules scattered throughout the peritoneum, with serosal thickening (Munday et al., 2017; Uzal et al., 2016). Histologically, it is classified into three types: epithelioid, sarcomatous (fibrous), or mixed (biphasic) (Caswell & Williams, 2016; Munday et al., 2017).

In felines, mesothelioma more frequently affects the pleura and is less often diagnosed in the peritoneum (Munday et al., 2017). The most common clinical signs include difficulty breathing due to the accumulation of fluid in the pleura or abdominal distension caused by the accumulation of fluid in the peritoneum, resulting from exudation from the tumor surface or lymphatic vessels obstructed by the tumor (Garrett, 2007). Patients are usually euthanized following diagnosis due to a poor prognosis (Morris & Dobson, 2001).

Given the importance of oncological cases in veterinary medicine, here, we report an atypical case of metastatic peritoneal mesothelioma in a feline and discuss the macroscopic, microscopic, and immunohistochemical findings.

## Case description

A female, 2-year-old mixed-breed cat was referred to the Veterinary Hospital of the Universidade Federal de Uberlândia (HOVET-UFU) for a necropsy examination. The animal had previously been treated in a private clinic. The patient's complete medical record was not provided. In the clinical record sent with the feline, moderate abdominal distension was described as the only clinical sign. Abdominal ultrasound examination revealed moderate peritoneal effusion. A cytological examination of the fluid from the abdominal cavity was performed, leading to the provisional diagnosis of mesothelioma/carninomatosis. Subsequently, the animal was sent for exploratory laparotomy and tissue collection for histopathological examination. Although the patient apparently recovered well after surgery, he died the following day and was sent to the Animal Pathology Laboratory at HOVET-UFU.

**Figure 1:**
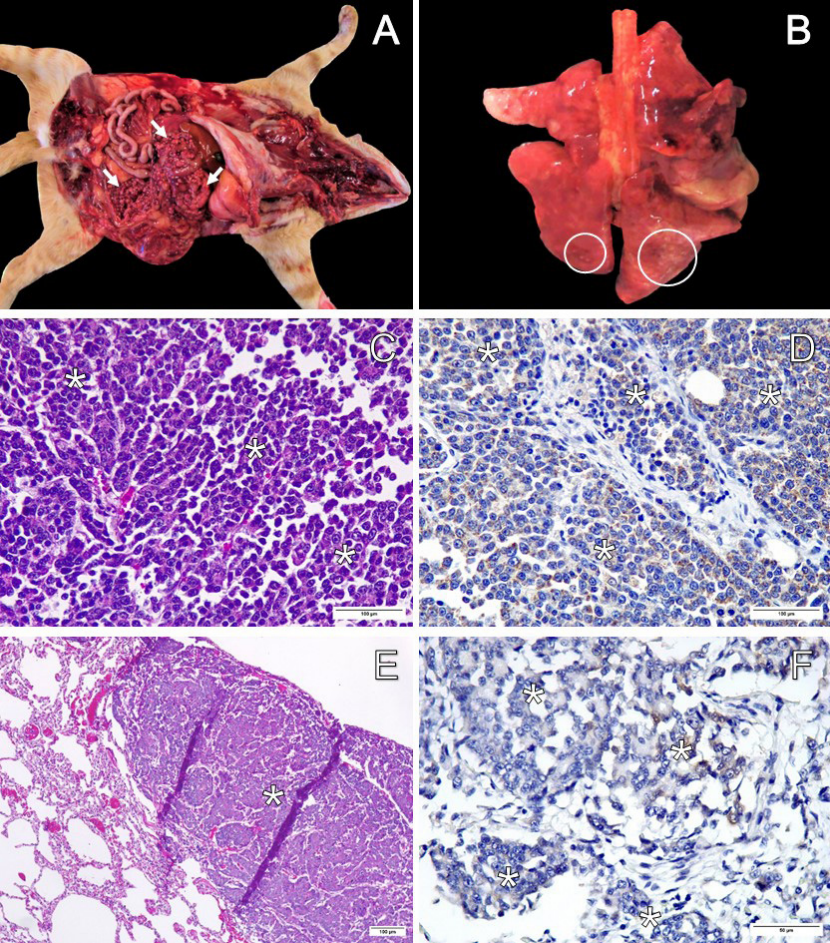
Feline, female, mixed breed, 2 years old, diagnosed with solid epithelioid peritoneal mesothelioma, through histopathology and immunohistochemistry. (A) Macroscopic photo of the opening of the abdominal cavity and exposure of the abdominal organs, demonstrating the omentum with multiple nodules, diffuse, sometimes coalescing, measuring 0.2 to 2 cm, firm, whitish, and irregular (arrows); (B) Macroscopic photo showing caudal lung lobes with multiple whitish, firm, millimetric nodules in the parenchyma (circles); (C) Photomicrograph of one of the nodules in the omentum showing proliferation in a solid arrangement (asterisks) of rounded to oval cells with moderate and eosinophilic cytoplasm, evident nucleolus, and moderate cellular pleomorphism. Suggestive of solid epithelioid peritoneal mesothelioma (H.E., 20x objective, 100 μm scale); (D) Anti-calretinin immunohistochemical analysis of the anterior nodule (C) showing neoplastic cells with strong cytoplasmic immunostaining (asterisks). Indicative of solid epithelioid peritoneal mesothelioma (20x objective, scale 100 μm); (E) Photomicrograph of a lung fragment with neoplastic proliferation similar to that observed in the histopathology of nodules in the omentum, characterizing metastasis of peritoneal mesothelioma (asterisk) (H.E., 10x objective, scale 100 μm); (F) Anti-calretinin immunohistochemical analysis of the previous fragment (E) showing weak cytoplasmic immunostaining (asterisks). Indicative of peritoneal mesothelioma metastasis (40x objective, scale 50 μm).

During the necropsy, a body condition score of 7 was observed (Laflamme, 1997). In the abdominal cavity, 22.0 mL of thick, dark red liquid was collected. The liver was extremely pale, with a yellowish color and an evident lobular pattern, but without evidence of neoformation, as described in the previous examination. Furthermore, in the peritoneum, diaphragm, omentum, stomach serosa, and large intestine, multiple nodules ranging from 0.2 to 2.0 cm in diameter, coalescent, firm, whitish, and with an irregular surface were observed (Figure 1A). When cut, these nodules were homogeneous and whitish.

In the thoracic cavity, there was 6.0 mL of reddish watery fluid, and the lung parenchyma showed multiple millimeter-sized, firm, and whitish nodules, distributed diffusely throughout the organ (Figure 1B). The lymph nodes in the pleural and abdominal cavities showed no macroscopic changes.

Nodule samples from the peritoneum, diaphragm, omentum, serosa (stomach and large intestine), and lungs were collected, fixed in 10% buffered formalin, sectioned, embedded in paraffin, cut into 5-μm sections, stained with hematoxylin and eosin, and examined under an optical microscope. Additionally, nodule samples from the omentum and lungs were subjected to immunohistochemical analysis, using anti-vimentin antibodies (Genemed Biotechnologies®️) at a 1:100 dilution, anti-cytokeratin AE1/AE3 antibodies (Dako®️) at a 1:400 dilution, and anti-calretinin antibodies (Spring Bioscience®️) at a 1:200 dilution. The streptavidin-biotin-peroxidase complex (Dako®️) and diaminobenzidine (DakoCytomation®️) were used for antigen detection. Immunostaining was classified based on intensity as strong, moderate, weak, focal, and negative, and based on the distribution as 0%, < 25%, 25-75%, and > 75% of the tissue fragment (Bacci et al., 2006).

Microscopically, the nodule samples from the peritoneum, diaphragm, omentum, serosa (stomach and large intestine), and lungs were characterized by the proliferation of round to oval cells arranged in a solid pattern. These cells had a moderate, slightly eosinophilic cytoplasm, a round, centrally located nuclei with loose chromatin, evident nucleoli, sometimes multiple, and displayed moderate cellular pleomorphism, marked by moderate anisocytosis and anisocariosis. On average, there were seven to eight mitotic figures in the high-power field. A suggestive diagnosis of solid epithelioid peritoneal mesothelioma was assigned (Figure 1C).

An immunohistochemical panel was performed for cytokeratin AE1/AE3, vimentin, and calretinin for the definitive diagnosis of mesothelioma. Regarding AE1/AE3 anti-cytokeratin antibody immunostaining, samples from the omental nodules showed strong cytoplasmic immunostaining in > 75% of the fragment, whereas the pulmonary nodules demonstrated weak cytoplasmic immunostaining in < 25% of the fragment. There was no immunoreactivity for both samples with the anti-vimentin antibody. As for the anti-calretinin antibody, strong cytoplasmic immunostaining was observed in > 75% of the fragment for the omental nodules (Figure 1D).

Lung metastasis was identified after histopathological analysis (Figure 1E), and failed cytoplasmic immunostaining for anti-calretinin antibodies was observed in < 25% of the lung fragment (Figure 1F). Histopathological and immunohistochemical examinations revealed that it was a case of solid epithelioid peritoneal mesothelioma with pulmonary metastasis.

## Discussion

Over the past 45 years, only nine cases of malignant feline peritoneal mesothelioma have been reported (Akiyama et al., 1982; Bacci et al., 2006; Heerkens et al., 2011; Kobayashi et al., 1994; Raflo & Nuernberger, 1978; Schlueter et al., 2021; Spugnini et al., 2008; Umphlet & Bertoy, 1988). Although the macro- and microscopic findings presented here coincide with previous descriptions, it is notable that, unlike the cases mentioned with ages ranging from 4 to 17 years, the animal in this report was younger (2 years), which represents a singular aspect in this case.

In the feline described here, the only clinical sign was abdominal distension, characterized by the presence of a free fluid in the peritoneal cavity, observed on ultrasound examination and subsequently confirmed at necropsy. Clinical signs in animals with peritoneal mesothelioma include weight loss and lethargy. As it is a highly effusive tumor, peritoneal effusion causes abdominal distension due to ascites (Heerkens et al., 2011; Morini et al., 2006; Morris & Dobson, 2001; Munday et al., 2017; Uzal et al., 2016). However, peritoneal effusions caused by mesotheliomas must be differentiated from peritonitis and, in cats, feline infectious peritonitis (Morris & Dobson, 2001).

Although the cytological analysis of fluids facilitates the differentiation between ascites of inflammatory or neoplastic origin (Munday et al., 2017), in cases of suspected mesothelioma, it is extremely challenging to differentiate reactive mesothelial cells from neoplastic ones (Morris & Dobson, 2001; Munday et al., 2017).

In the histopathological examination of the nodules in the peritoneum, diaphragm, omentum, serosa (stomach and large intestine), and lungs in this case, neoplastic proliferation of mesothelial cells forming solid aggregates was observed, compatible with epithelioid-type mesothelioma with pulmonary metastasis. The epithelioid subtype is the most common one in cats (Bacci et al., 2006; Heerkens et al., 2011; Weiss et al., 2010) and can be characterized by the presence of rounded cells with eosinophilic cytoplasm, anisokaryosis, evident nucleoli, or even multinucleated cells (Uzal et al., 2016).

When evaluating the metastasis in this case, the presence of pulmonary nodules and the absence of macroscopic changes in the thoracic and abdominal lymph nodes suggested that metastasis had occurred hematogenously. Although the etiology of peritoneal and retroperitoneal tumors remains uncertain (Caswell & Williams, 2016; Munday et al., 2017), some fiber types can develop mesotheliomas, mainly due to the size and solubility of such elements (Uzal et al., 2016).

It is essential to be aware of the fact that the histological components of mesotheliomas can vary from purely epithelial patterns to purely mesenchymal ones, and they can even be confused with primary carcinomas or sarcomas invading the peritoneal cavity (Munday et al., 2017). Metastases to serous membranes are common, macroscopically characterized by whitish plaques or even diffuse thickening areas, which can lead to an erroneous diagnosis of mesothelioma (Uzal et al., 2016). Furthermore, distinguishing between mesothelioma and activated or hyperplastic mesothelium is a tremendous challenge (Uzal et al., 2016). However, in this case, no macroscopic neoplastic changes were observed in the abdominal organs that would lead to the suspicion of primary carcinomas of the digestive, genital, or urinary tract. The affected feline presented nodules restricted to the omentum and serous membranes of the affected abdominal organs.

The definitive diagnosis of mesothelioma is challenging, and often, more specific tests, such as immunohistochemical panels, are required (Bacci et al., 2006). In this report, three antibodies were tested on samples collected from the omentum and lungs, namely anti-cytokeratin AE1/AE3, anti-vimentin, and anti-calretinin.

For cytokeratin, AE1/AE3, strong cytoplasmic immunostaining was observed in > 75% of the omentum fragment and weak cytoplasmic immunostaining in < 25% of the pulmonar fragment. Despite the scarcity of information on the immunohistochemical characteristics of mesothelioma in domestic animals (Piacenti et al., 2004), immunostaining for cytokeratin and vimentin aids in differentiating mesothelioma from epithelial or non-epithelial neoplasms (Caswell & Williams, 2016). Additionally, it is possible to distinguish peritoneal sarcomas, which do not show immunostaining for cytokeratin (Munday et al., 2017). There was no immunoreactivity for vimentin, and a possible explanation for this could be the differentiation of neoplastic mesothelial cells into a purely epithelial pattern, characterizing an epithelioid-type mesothelioma.

Due to immunostaining only for cytokeratin AE1/AE3, immunohistochemistry for calretinin was performed for differential diagnosis. A study conducted on 244 cases of human peritoneal mesothelioma demonstrated that calretinin is a sensitive mesothelial marker for the immunohistochemical evaluation of such neoplasms (Tandon et al., 2018). Strong cytoplasmic immunostaining was observed in > 75% of the omentum fragment, and weak immunostaining in < 25% of the lung fragment. Calretinin is a calcium-binding protein expressed in various tissues, including normal and neoplastic mesothelium (Cury et al., 2000; Doglioni et al., 1996; Lugli et al., 2003). In the report of a primary diffuse malignant epithelioid peritoneal mesothelioma in a striped skunk (*Mephitis mephitis*), there was strong immunostaining for calretinina (Kim et al., 2016).

## Conclusion

In conclusion, the presented case of metastatic peritoneal mesothelioma in a 2-year-old feline underscores the rarity and diagnostic challenges associated with this condition in veterinary medicine. The age variation observed in this case compared to documented cases adds a unique dimension to our understanding. Comprehensive examination, including macroscopic, microscopic, and immunohistochemical analyses, not only confirmed the diagnosis but also highlighted the importance of such detailed approaches in discerning mesothelioma from other neoplasms. This atypical presentation serves as a reminder of the complexities involved in diagnosing and managing oncological cases in veterinary practice.
